# A comparative study of risk factors in predictive models for cognitive dysfunction in patients with leukoaraiosis based on machine learning algorithms

**DOI:** 10.3389/fnins.2025.1654584

**Published:** 2025-12-10

**Authors:** Wenwen Xu, Jianning Li, Chunfang Huang, Yi Lu, Liping Yin, Guoxin Zhang

**Affiliations:** 1 Qixia District Hospital, Nanjing, Jiangsu, China; 2 Nanjing Lishui People's Hospital, Zhongda Hospital, Southeast University, Nanjing, China

**Keywords:** leukoaraiosis, cognitive dysfunction, machine learning, predictive model, risk factors

## Abstract

**Objective:**

To explore the risk factors of cognitive dysfunction in patients with leukoaraiosis (LA) and to construct a predictive model using machine learning.

**Methods:**

A total of 273 patients with LA were included. Univariate analysis and multivariate logistic regression were performed to identify independent risk factors for cognitive dysfunction. The patients were divided into a training set (191 cases) and a validation set (82 cases) in a 7:3 ratio. Seven machine learning algorithms (Decision Tree, GBDT, Logistic Regression, Random Forest, SVM, KNN, XGBoost) were employed to construct predictive models. Evaluation metrics included accuracy, recall, F1 score, MCC, AUROC, and SHAP was used for model interpretation.

**Results:**

Univariate analysis revealed that age, LDL-C, uric acid, CRP, Fazekas score, and IASA score were associated with cognitive dysfunction (*p* < 0.05). Multivariate logistic regression analysis showed that age, LDL-C, uric acid, Fazekas score, and IASA score were independent risk factors (*p* < 0.05). Among the machine learning algorithms, the Random Forest model performed the best, with an AUROC of 0.8373 for the validation set. SHAP analysis indicated that age, LDL-C, IASA score, and Fazekas score were the most important predictors.

**Conclusion:**

The Random Forest model can be used to predict the risk of cognitive dysfunction in patients with LA, providing a reference for early warning.

## Background

Leukoaraiosis (LA), also known as White Matter Hyperintensities (WMH), is a common form of cerebral small vessel disease that is widely present in the elderly population (Tang et al., 2021; Hentabli et al., 2022). The clinical symptoms of LA are subtle and non-specific in the early stages, and as the condition progresses, a series of clinical issues gradually emerge, including cognitive impairment, dementia, gait abnormalities, and emotional confusion (Kongbunkiat et al., 2017). Multiple studies have shown that LA has become an important risk factor for frailty, decline in quality of life, and even death in the elderly (Chae et al., 2020; Xu et al., 2023). Cognitive impairment caused by LA is often overlooked in its early stages, and by the time it is diagnosed, most patients already have moderate to severe cognitive impairment or even dementia (Schmidt et al., 2007). This

limits subsequent treatment options and reduces their effectiveness, placing a heavy mental and economic burden on the families of patients and even exacerbating social conflicts (Chojdak-Łukasiewicz et al., 2021). The mechanisms underlying the development of LA are not fully understood, and no single theoretical explanation can comprehensively account for its formation (Sun et al., 2022). Under the premise of managing traditional risk factors, regular monitoring of biological indicators and imaging features, along with adjustments in lifestyle and medical treatments to control abnormal indicators (Feng and Yan, 2022), can help delay the progression of LA and reduce the rate of conversion from mild cognitive impairment to dementia in LA patients (Rastogi et al., 2022).

In recent years, the complexity of clinical data has increased, and traditional statistical methods face certain limitations in exploring and mining large sample, multi-variable data (Huo et al., 2021). The mechanisms underlying cognitive dysfunction caused by LA are complex, with diverse influencing factors, necessitating new approaches. Machine learning (ML), a powerful data analysis tool in artificial intelligence (AI), has rapidly developed in the medical field (Karvelas and Elahi, 2023). Initially applied to commercial data analysis, machine learning's ability to process complex and dynamic data has unlocked great potential for understanding disease patterns, diagnosis, and treatment (Verhaaren et al., 2015; Lee et al., 2021). The interactions between clinical variables are intricate, and using machine learning to build predictive models can better understand and predict clinical events. Machine learning-based models enhance the ability to handle common variables faced by traditional statistical methods and can fully account for non-linear relationships, thus improving the applicability and accuracy of clinical predictions. Supervised learning algorithms and unsupervised learning are the main components of machine learning (Tubi et al., 2020). Common supervised learning algorithms include Random Forest, Support Vector Machine (SVM), K-nearest neighbors, neural networks, and Naive Bayes (Dickie et al., 2020; Desmarais et al., 2021). Common unsupervised learning algorithms include clustering algorithms, dimensionality reduction methods, and association rule learning (Hase et al., 2018).

Early-stage LA patients do not exhibit obvious clinical symptoms, and early diagnosis of cognitive impairment in LA patients is crucial for assisting clinicians in diagnosis and improving patient prevention and treatment plans (Filley and Fields, 2016). Unfortunately, most previous studies have focused on a single type of risk factor, which significantly limits predictive effectiveness (Zeng et al., 2020). Although some studies have performed multivariable analyses, the results often appear too complex, or their accuracy is difficult to compare and evaluate intuitively (Morley, 2015; Bender and Raz, 2015). Establishing a simple, sensitive, and efficient predictive model is of great clinical value for the early identification and prevention of high-risk groups of cognitive impairment in LA patients (van Gijn, 1998). Therefore, constructing and selecting an efficient predictive model based on machine learning technology is of significant importance for accurately predicting cognitive impairment in LA patients, and it is also a primary task in current primary prevention efforts (Carnevale et al., 2018; Gómez-Soria et al., 2023). This study uses machine learning algorithms to construct a predictive model aimed at finding a high-precision, multidimensional, high-sensitivity, and specificity machine learning model for early prediction of cognitive dysfunction in LA patients, enabling classification and identification of high-risk populations (Yuan et al., 2021; Sakurai et al., 2015; Fu et al., 2020). This can reduce the risk of LA patients further developing into dementia, decrease medical costs, improve patient prognosis, and ultimately enhance patients' quality of life (Zhang et al., 2022; Choi et al., 2020).

## Methods

### Study subjects

This study used data from the Cerebral Small Vessel Disease (CSVD) research project (NQH-CSVDrp) at Qixia District Hospital, Nanjing, China, which began in June 2020. The project aims to identify biomarkers for early cognitive impairment detection in CSVD, predict cognitive decline progression, and offer personalized interventions to prevent vascular dementia. By April 2023, 390 CSVD patients were included, all providing informed consent, and the study was approved by the hospital's ethics committee. Demographic data and medical history were recorded, including age, gender, marital status, education level, BMI, anxiety, socioeconomic status, and income level. Personal and past medical history: smoking history, alcohol consumption history, hypertension, type 2 diabetes, coronary heart disease, etc.

#### Inclusion criteria

(1) Imaging characteristics consistent with LA: high signal lesions on T2 FLAIR, low signal on T1, and rare infarctions in the brainstem, thalamus, and basal ganglia. (2) Complete medical records and cooperation with the study. (3) Age between 50 and 85 years.

#### Exclusion criteria

(1) History of moderate to severe stroke (NIHSS ≥5). (2) White matter degeneration due to hereditary or inflammatory diseases. (3) White matter changes from toxic, immunological, or other causes. (4) Severe heart, lung, or liver diseases. (5) Cognitive impairment due to metabolic or infectious diseases. (6) Death during hospitalization. (7) Missing data >20%. (8) Co-existing neurodegenerative diseases, such as Parkinson's or Alzheimer's disease.

### Laboratory tests

Serum blood markers were recorded within 24 hours of admission after an overnight fast, including triglycerides, total cholesterol, low-density lipoprotein, uric acid, plasma lipoprotein-associated phospholipase A2, fasting blood glucose, urinary albumin/creatinine ratio, thyroid hormone, serum creatinine, C-reactive protein, total bilirubin, urea, and homocysteine.

### Neurological scale assessment

Cognitive function was assessed by neurologists trained in standardized protocols. Cognitive assessment: The Mini-Mental State Examination (MMSE) was used as a basis to evaluate cognitive function in enrolled participants. Detailed scores for each item were recorded, with a total score of 30 points. Participants with a score ≤ 24 were classified as having cognitive impairment. For participants with zero years of education (illiterate), 6 points were added to the total score to correct for educational bias. For participants with ≤ 6 years of education, 4 points were added to correct for educational bias. Based on the MMSE score, participants were divided into the cognitive impairment (CI) group and non-cognitive impairment (NCI) group.

MMSE, a widely used tool for assessing cognitive function, covers five domains with a total score of 30. Scores reflect mental status: 26–30 indicates normal cognition with no significant impairment; 21–25 suggests mild dementia with possible slight cognitive difficulties in daily life; 10–20 indicates moderate dementia with noticeable cognitive impairment in multiple areas; and 0–9 signifies severe dementia, with significant cognitive impairment across multiple domains, requiring substantial care and support (Folstein et al., 1975).

NIHSS is a standardized tool for assessing neurological deficits in stroke patients. It includes 15 evaluation items, with scores reflecting neurological function—lower scores indicate better status (Brott et al., 1989).

### Imaging assessment

#### LA severity grading and location (Fazekas score)

MRI high-signal manifestations of periventricular and deep white matter on T2 images were assessed using the Fazekas scale. The scores from both sections were summed to determine the severity level. Periventricular: No abnormalities (0 points); Spot-like changes (1 point); Fused lesions (2 points); Large fused lesions (3 points). Deep white matter: No abnormalities (0 points); Pencil-like or cap-like changes (1 point); Halo-like changes (2 points); Irregular changes extending into the deep white matter (3 points). The total sum of both scores determines the severity grading of LA (Fazekas et al., 1987).

#### Intracranial artery stenosis assessment (IASA)

MRA scanning was performed using the Neusoft NSM-S15P 1.5T, TOF_3D_MRA sequence, with a 16-channel brain coil. The scanning parameters were set to TR 22ms, TE 6.9ms, flip angle < 20.1°, and matrix size 256 × 256. A total of 161 cross-sectional images were collected, with a scanning average time of 3.34 minutes. The collected images were processed for three-dimensional vascular reconstruction. The major intracranial and extracranial vessels, including the bilateral internal carotid arteries, anterior cerebral artery, middle cerebral artery, posterior cerebral artery, basilar artery, and vertebral arteries, were primarily assessed. The degree of vascular stenosis was calculated as: Where DstenosisD_Dstenosis is the diameter of the vessel at its most narrowed point, and DnormalD is the diameter of the normal proximal vessel. If multiple stenoses are present, the most severe stenosis is used for calculation. The severity of vascular stenosis was classified into 3 levels: (1) Any vessel with stenosis < 20% is assigned 2 points. (2)Two or more vessels with stenosis > 40% are assigned 4 points. (3) Stenosis between these two levels, indicating mild stenosis, is assigned 3 points. (4) No stenosis is assigned 1 point.

### Machine learning model construction

Missing data were assessed before analysis. Variables with less than 10% missing values were imputed using the mean or mode, while those with more than 20% missing data were excluded. Multiple imputation was applied for variables with 10–20% missingness.

### Logistic regression model

Logistic regression is commonly used in binary and multiclass classification tasks, especially in medical statistics. The Sigmoid function is used to map a linear combination of input features to probabilities. The goal is to model the probability of the dependent variable, not the variable itself.

### Random forest model

Random Forest is an ensemble learning method that combines multiple weak models into a strong one. Key steps include: Bootstrap aggregation: Random sampling with replacement to create independent training datasets. Decision trees: Trees are built on different subsets, selecting features randomly for splits. Overfitting reduction: Different training datasets reduce overfitting risk.

### SVM model

SVM uses nonlinear mapping to project data into higher-dimensional feature spaces for better classification. It finds the optimal decision boundary that maximizes the margin between classes. SVM is effective for small sample tasks and nonlinear problems. Kernel functions avoid the curse of dimensionality, enabling efficient learning in high-dimensional spaces.

### BP neural network model

The BP neural network is a multi-layer model that simulates the human brain's processing. It adjusts weights and thresholds through gradient descent to minimize errors. It consists of an input layer, hidden layers, and an output layer. BP networks are highly adaptable and effective for large-scale, complex data analysis.

### Statistical analysis methods

Statistical analysis was performed using SPSS 22.0. Continuous variables were described as mean ± standard deviation or median (interquartile range), and group comparisons were made using t-tests or Mann-Whitney U tests. Categorical variables were described as counts (percentages), and group comparisons were performed using chi-square tests. Variables with *P* < 0.05 in univariate analysis were included in the multivariate logistic regression model to calculate the odds ratio (OR) and 95% confidence interval (CI). A *p* value < 0.05 was considered statistically significant. Machine learning was implemented using Python 3.7 and R 4.0.1, including decision trees, GBDT, logistic regression, random forests, SVM, KNN, and XGBoost. Evaluation metrics included accuracy, recall, F1 score, MCC, and AUROC. SHAP was used for model interpretation.

## Results

### Baseline characteristics of the training cohort

A total of 273 patients with LA were included in this study, which were divided into a training set (191 cases) and a validation set (82 cases) at a 7:3 ratio. Table 1 shows the baseline characteristics of the training set population. Among the 191 patients, 88 were male (46.07%) and 103 were female (53.93%). The results indicated that there were statistically significant differences in age, low-density lipoprotein cholesterol (LDL-C), uric acid (UA), C-reactive protein (CRP), Fazekas score, and IASA score (P < 0.05). These differences suggest that clinical and biochemical indicators in the patient population exhibit certain characteristic variations, which may influence the occurrence and development of LA.

**Table 1:** Description of the training set population.

**Variable Names**	**Overall**	**0**	**1**	***p***
	*N* = 191	*N* = 102	*N* = 89	
15.6-2.2,-1.3498ptAge	74.64 ± 7.82	71.75 ± 7.58	77.97 ± 6.72	< 0.01
**Sex (%)**				0.31
Female	103 (53.93)	59 (57.84)	44 (49.44)	
15.6-2.2,-1.3498ptMale	88 (46.07)	43 (42.16)	45 (50.56)	
**Hypertension (%)**				0.10
Yes	136 (71.20)	67 (65.69)	69 (77.53)	
15.6-2.2,-1.3498ptNo	55 (28.80)	35 (34.31)	20 (22.47)	
**T2DM (%)**				0.39
Yes	70 (36.65)	34 (33.33)	36 (40.45)	
15.6-2.2,-1.3498ptNo	121 (63.35)	68 (66.67)	53 (59.55)	
**Smoking (%)**				0.67
Yes	54 (28.27)	27 (26.47)	27 (30.34)	
15.6-2.2,-1.3498ptNo	137 (71.73)	75 (73.53)	62 (69.66)	
**Drinking (%)**				1.00
Yes	41 (21.47)	22 (21.57)	19 (21.35)	
No	150 (78.53)	80 (78.43)	70 (78.65)	
Triglyceride	2.21 ± 1.53	2.36 ± 1.61	2.03 ± 1.43	0.14
Total_Cholesterol	3.56 ± 1.57	3.48 ± 1.59	3.66 ± 1.55	0.42
LDL	2.08 ± 0.85	1.95 ± 0.75	2.22 ± 0.92	0.03
UA	305.39 ± 110.59	281.24 ± 100.17	333.07 ± 115.93	< 0.01
ALp_PLA2	188.38 ± 73.85	186.94 ± 72.2	190.03 ± 76.06	0.77
Glucose	6.36 ± 2.08	6.26 ± 1.9	6.48 ± 2.27	0.47
ACR	4.06 ± 8.34	3.75 ± 7.59	4.41 ± 9.16	0.59
TSH	3.59 ± 3.91	3.67 ± 4.38	3.49 ± 3.32	0.76
Creatinine	84.63 ± 93.82	79.36 ± 92.67	90.66 ± 95.29	0.41
CRP	6.47 ± 14.46	4.13 ± 7.03	9.16 ± 19.53	0.02
TBIL	15.02 ± 8.89	14.01 ± 7.38	16.17 ± 10.28	0.09
Urea	5.76 ± 3.21	5.82 ± 4	5.69 ± 1.98	0.78
15.6-2.2,-1.3498ptHomocysteine	14.76 ± 10.74	14.97 ± 14.15	14.52 ± 4.41	0.78
**IASA (%)**				< 0.01
1	98 (51.31)	67 (65.69)	31 (34.83)	
2	54 (28.27)	27 (26.47)	27 (30.34)	
3	29 (15.18)	6 (5.88)	23 (25.84)	
15.6-2.2,-1.3498pt4	10 (5.24)	2 (1.96)	8 (8.99)	
**Fazekas score (%)**				< 0.01
1	67 (35.08)	55 (53.92)	12 (13.48)	
2	69 (36.13)	36 (35.29)	33 (37.08)	
3	32 (16.75)	10 (9.80)	22 (24.72)	
4	11 (5.76)	0 (0.00)	11 (12.36)	
5	12 (6.28)	1 (0.98)	11 (12.36)	

### Multivariate analysis of cognitive dysfunction in patients with white matter leukoaraiosis

**Figure 1:**
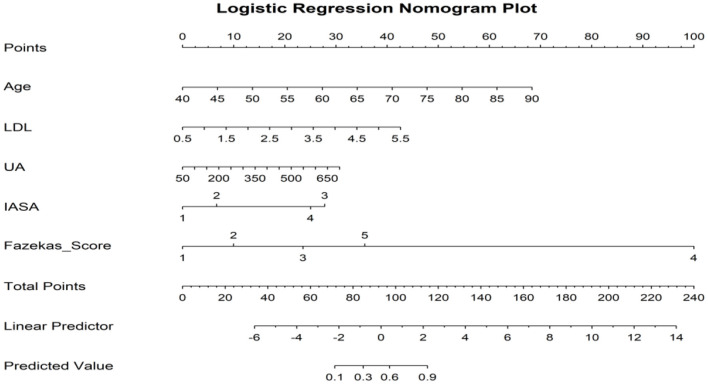
A nomogram of risk factors for cognitive dysfunction in patients with LA. When using the nomogram to predict the risk of cognitive impairment, the specific value of each variable was first found on the corresponding coordinate axis, and then a vertical line was drawn up to the Points axis to determine the score of the variable. The predicted Risk of leukoaraiosis can be obtained by summing the scores of all variables to obtain the total score, and finally finding the corresponding total score on the risk axis at the bottom of the figure.

The variables with *p* < 0.05 in the univariate analysis were included in the binary logistic regression model, and the forward conditional method was used to select the variables. The results showed that age, LDL-C, UA, Fazekas score, and IASA score are independent risk factors for LA (*p* < 0.05). See Table 2 for details. To visually display the relationship between each risk factor and the risk of white matter leukoaraiosis, a nomogram based on the logistic regression coefficients was created (Figure 1).

**Table 2:** The results of the Logistic regression analysis for cognitive dysfunction in patients with LA.

**Variable name**	**Estimate**	**Std_ Error**	**Z_ value**	***P* value**	**OR (95% CI)**
(Intercept)	−17.1726	2.9085	−5.9043	-	-
Age	0.1291	0.0316	4.0839	0.0000	1.1378 (1.0733–1.2155)
LDL	0.7373	0.2576	2.8627	0.0042	2.0903 (1.2874–3.5521)
UA	0.0043	0.0019	2.2119	0.0270	1.0043 (1.0006–1.0082)
CRP	0.0377	0.0207	1.8178	0.0691	1.0384 (1.0036–1.0878)
IASA	1.0783	0.2615	4.1242	0.0000	2.9398 (1.8023–5.0628)
Fazekas_Score	1.1825	0.2444	4.8383	0.0000	3.2625 (2.0839–5.4668)

### Building a predictive model based on machine learning

**Figure 2:**
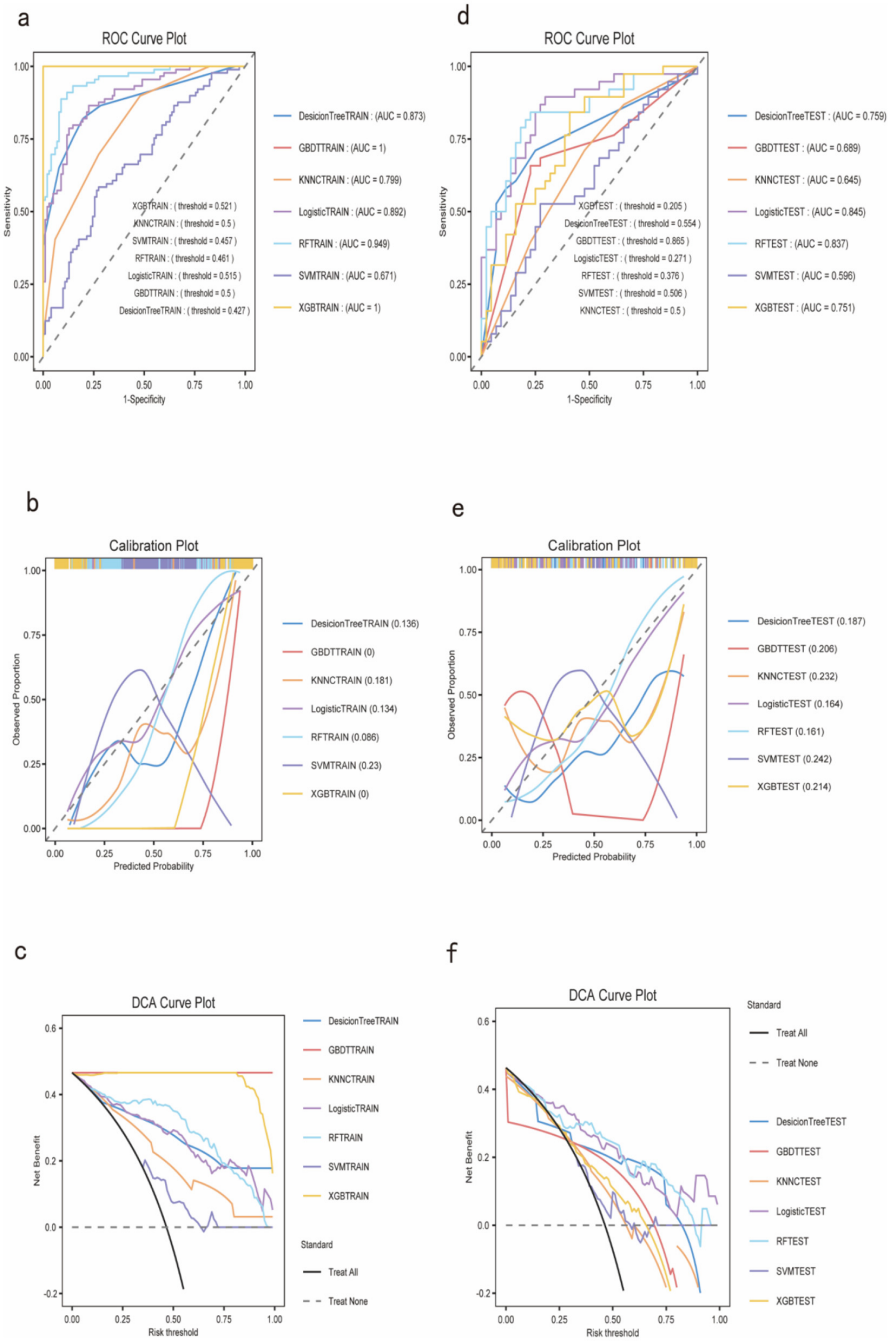
Performance evaluation of machine learning models. **(a)** ROC curves for the training set; **(b)** Calibration plot for the training set; **(c)** DCA curve for the training set; **(d)** ROC curves for the validation set; **(e)** Calibration plot for the validation set; **(f)** DCA curve for the validation set.

To construct a more accurate predictive model for cognitive dysfunction in LA, this study attempted to use machine learning algorithms. We employed seven common classification algorithms, including Decision Tree, Gradient Boosting Decision Tree (GBDT), Logistic Regression, Random Forest (RF), Support Vector Machine (SVM), K-Nearest Neighbors (KNN), and Extreme Gradient Boosting (XGB). Tables 3, 4 show the performance metrics of each model on the training and validation sets, respectively. On the training set, GBDT and XGB achieved the best performance, with accuracy, recall, and other metrics reaching 100%, followed by Random Forest (88.48%). However, on the validation set, the Random Forest model demonstrated the most robust performance, with an accuracy of 78.05% and an AUROC of 0.8373, significantly outperforming the other models. Therefore, we selected Random Forest as the final predictive model. The DCA curve, ROC curve, and decision curve for the training and validation sets in Figure 2 visually demonstrate the superiority of the Random Forest model.

**Table 3:** Performance indicators of 7 models in the training set (SD).

**Model name**	**Accuracy**	**Recall**	**F1-Score**	**MCC**	**AUROC**	**Precision**	**Specificity**	**FNR**
Decision tree TRAIN	0.8115	0.8202	0.8022	0.6229	0.8728	0.7849	0.8039	0.1798
GBDTTRAIN	1.0	1.0	1.0	1.0	1.0	1.0	1.0	0.0
Logistic TRAIN	0.8272	0.7865	0.8092	0.6525	0.8923	0.8333	0.8627	0.2135
RFTRAIN	0.8848	0.8539	0.8736	0.7686	0.9488	0.8941	0.9118	0.1461
SVMTRAIN	0.6230	0.3820	0.4857	0.2428	0.6711	0.6667	0.8333	0.6180
KNNCTRAIN	0.7120	0.6966	0.6927	0.4218	0.7991	0.6889	0.7255	0.3034
XGBTRAIN	1.0	1.0	1.0	1.0	1.0	1.0	1.0	0.0
Mean scores	0.8369	0.7913	0.8091	0.6727	0.8834	0.8383	0.8768	0.2087

**Table 4:** Performance indicators of seven models in the validation set (SD).

**Model name**	**Accuracy**	**Recall**	**F1-Score**	**MCC**	**AUROC**	**Precision**	**Specificity**	**FNR**
KNNCTEST	0.6098	0.7105	0.6279	0.2361	0.6450	0.5625	0.5227	0.2895
SVMTEST	0.5976	0.4474	0.5075	0.1821	0.5963	0.5862	0.7273	0.5526
RFTEST	0.7805	0.6842	0.7429	0.5600	0.8373	0.8125	0.8636	0.3158
Logistic TEST	0.7683	0.6842	0.7324	0.5340	0.8451	0.7879	0.8409	0.3158
GBDTTEST	0.7073	0.6579	0.6757	0.4099	0.6890	0.6944	0.75	0.3421
Decision Tree TEST	0.7317	0.6053	0.6765	0.4619	0.7593	0.7667	0.8409	0.3947
XGBTEST	0.6463	0.6053	0.6133	0.2877	0.7512	0.6216	0.6818	0.3947
mean_ scores	0.6916	0.6278	0.6537	0.3817	0.7319	0.6903	0.7468	0.3722

### Random forest SHAP interpretation

**Figure 3:**
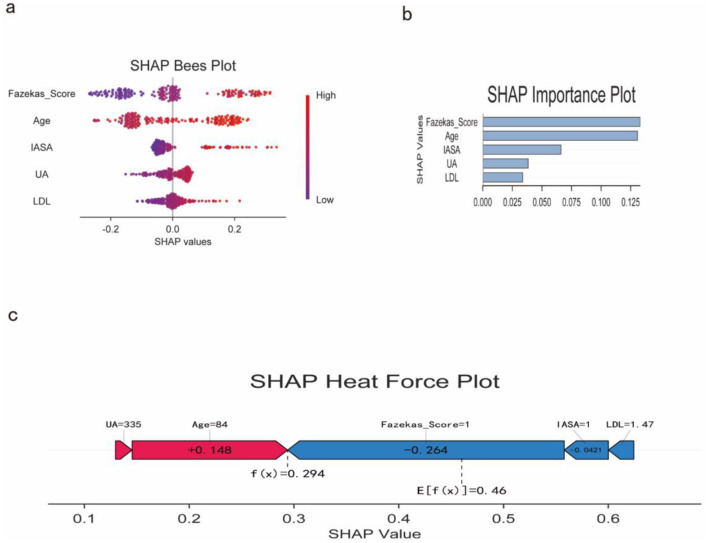
SHAP analysis of the Random Forest model. **(a)** SHAP bees plot showing the distribution and impact of each feature on model predictions; **(b)** SHAP importance plot showing mean absolute SHAP values for each feature; **(c)** SHAP force plot for a representative patient demonstrating individual feature contributions.

To explain the predictive mechanism of the random forest model, we performed SHAP analysis. The SHAP swarm plot of Figure 3a reveals the direction (positive and negative) and magnitude of the influence of each feature on the model output. Figure 3b, sorted by mean absolute SHAP value magnitude, shows that age, LDC-C, IASA score, and Fazekas score are the most important predictors. Figure 3c is the SHAP chart of a typical patient, which intuitively shows the direction and contribution of each feature, among which age and Fazekas score have the greatest negative effect.

## Discussion

### Risk factors for cognitive dysfunction in LA

Previous studies have shown that age is one of the significant independent risk factors for LA (Heo et al., 2019; Fan, 2022). As age increases, there is a positive correlation with the quantity and density of WMH, as well as a parallel relationship with the severity of cognitive impairment (Chojdak-Łukasiewicz et al., 2021; Aspray and Hill, 2019). The potential mechanism by which age contributes to LA is primarily due to the natural aging of brain vessels, leading to a reduction in cerebral blood flow, ischemic and hypoxic changes in brain tissue, and the loss of the integrity of the white matter (Chen et al., 2019). In addition, the negative impact of cellular metabolism and oxidative stress associated with aging on the white matter should also be considered. These multiple factors lead to structural damage to the white matter, ultimately resulting in cognitive dysfunction in LA patients.

Intracranial arterial stenosis is an independent risk factor for LA-related cognitive impairment, and the degree of stenosis in intracranial arteries is proportional to the severity of cognitive dysfunction. Both domestic and international studies (Wang et al., 2018)suggest that patients with intracranial arterial stenosis have more severe LA, as the stenosis reduces the regulation ability of cerebral vessels, affecting hemodynamics and causing prolonged low blood perfusion in the brain. Under low perfusion conditions, the supply of oxygen and nutrients to the white matter is reduced, leading to damage of white matter fibers and a decline in the efficiency of brain network system connectivity, resulting in cognitive impairment. Intracranial arterial stenosis can also lead to the formation of microemboli, significantly increasing the overall incidence of stroke events. Acute stroke events trigger systemic changes in the brain, including the loss of white matter signal connections, neuroinflammatory reactions, and blood-brain barrier disruption, leading to damage to the brain's network system. Furthermore, during the chronic phase of a stroke, brain tissue may undergo secondary degeneration of white matter tracts and cortical atrophy, causing disruptions or even interruptions in the information connectivity of the frontal and temporal lobes, ultimately leading to cognitive damage. Liu et al. found that microvascular occlusion due to intracranial artery stenosis occurs in the white matter region, where this damage is more sensitive. Additionally, in a long-term observational study, Vincent Mok et al. found that patients with intracranial artery stenosis had a faster progression of WMH and a more significant decline in cognitive function compared to a control group. These studies collectively reveal that intracranial artery stenosis is a risk factor for cognitive impairment in LA, providing important theoretical support for clinicians in diagnosis and treatment.

The Fazekas scoring system was first developed in the 1990s by Fazekas et al., who used MRI imaging to score the periventricular and deep white matter lesions (Cedres et al., 2020). The traditional Van Swieten scale is limited to using CT imaging to assess the severity of LA, but MRI is far more sensitive than CT. Therefore, using the Fazekas score for assessing the severity of LA is considered more rigorous. The Fazekas score grades the appearance of WMH on MRI and separately scores periventricular and deep lesions before calculating the total score. This study found that the Fazekas score is positively correlated with age and shows a parallel relationship with the severity of cognitive dysfunction. A cross-sectional study by E. Labos et al. on MIC patients over the age of 65 who underwent head MRI found that the progression of structural damage to the white matter affected the correlation with overall cognitive and executive function. Moreover, Sandeepa Sur et al. also identified a significant association between the increase in the Fazekas score and the onset of mild Alzheimer's disease.

Therefore, the Fazekas score, as an effective tool, has significant value not only in clinical diagnosis but also in predicting the development of WMH and its associated cognitive impairment. As the score increases, the severity of WMH also increases, leading to further cognitive decline. The application of the Fazekas score deepens our understanding of age-related brain lesions and their impact on cognition, providing a foundation for targeted research and interventions.

### Construction of machine learning models

This study constructs four predictive models: BP neural network, Random Forest, Logistic Regression, and SVM models. Since LA is a disease caused by the combined effect of multiple factors, its impact on cognitive function involves a comprehensive range of biological markers and clinical indicators. The ability of machine learning models to handle high-dimensional data and simulate complex biological processes is significant, making them suitable for accurately predicting cognitive dysfunction in LA patients (Arfat et al., 2022). The ROC curve and calibration curve are used to evaluate and identify the ability to predict cognitive dysfunction, especially the area under the ROC curve (AUC), which is an important indicator of the model's diagnostic capability, reflecting the overall effect in terms of false positive and true positive rates (Wang et al., 2018). The calibration curve reflects the accuracy of the model's predicted probability, with a curve approaching the 45-degree diagonal indicating that the predicted probability is consistent with the actual occurrence probability. Based on the accuracy of different models in predicting the risk of cognitive dysfunction in LA patients, clinical decisions can adjust clinical decision thresholds to optimize the diagnostic and treatment pathways for patients (Sadatsafavi et al., 2022). The ROC curve provides a pure accuracy indicator by showing the limits of the test's ability to distinguish between different health states across the entire range of working conditions (Sadatsafavi et al., 2022). Moreover, the ROC curve plays a central or unified role in the evaluation and use of diagnostic tools, performing quantitative ROC analysis and testing comparisons, modifying individual subject disease probabilities using likelihood ratios, selecting decision thresholds, using logistic regression analysis, performing discriminant function analysis, or incorporating the tool into clinical strategies through decision analysis (Zweig and Campbell, 1993). In a cross-sectional study, the least absolute shrinkage and selection operator (LASSO) regression model and multivariate logistic regression analysis were used for variable selection to establish predictive models, and the predictive nomogram was developed and validated. The model was evaluated through the area under the ROC curve and a good calibration curve, and similar results were observed in 10-fold cross-validation, demonstrating the model's excellent discrimination ability (Huang et al., 2022).

### Comparison of predictive models

We used seven common classification algorithms, including Decision Tree, Gradient Boosting Decision Tree (GBDT), Logistic Regression, Random Forest (RF), Support Vector Machine (SVM), K-Nearest Neighbors (KNN), and Extreme Gradient Boosting (XGB). Random Forest, as the final predictive model, demonstrated the strongest stability in both the training and validation sets, significantly outperforming the other models. Ma et al. (2020) utilized MRI voxel-based morphometric measurements, deformation-based morphometric measurements, and surface-based morphometric measurements to extract morphological indicators such as gray matter volume, Jacobian determinant values, cortical thickness, gyrification index, sulcal depth, and fractal dimension (Duncan et al., 2020). They constructed a Random Forest predictive model and validated its performance through 10-fold cross-validation. Additionally, through the importance feature ranking in Random Forest, it was found that gyrification index and cortical thickness outperformed other features in identifying MCI, suggesting that they are key morphological biomarkers for early MCI diagnosis. Matthew Velazquez et al. used a balanced Random Forest model with clinical data to provide personalized predictions for the conversion from MCI to Alzheimer's Disease (AD) (Velazquez and Lee, 2021). They selected nine clinical features, including demographics, brain volume, and a mix of cognitive testing variables. The Random Forest model proved effective in predicting the conversion of EMCI patients to AD based on these clinical features (93.6% accuracy), serving as a tool for predicting the transition from the prodromal phase to AD or identifying ideal candidates for clinical trials. Furthermore, by evaluating the importance of each clinical feature through Random Forest, they excluded and focused on features that enhance interpretability.

The Random Forest model can be applied in clinical practice to assist physicians in early risk stratification and individualized treatment planning for patients with leukoaraiosis-related cognitive impairment. In addition, assessing its cost-effectiveness, implementation barriers, and integration into electronic health record systems will help determine the feasibility of incorporating this model into routine clinical workflows.

### Limitations

#### Limitations of sample size and data sources

The sample size and data sources used in this study may be limited, and the research primarily relies on specific populations (such as patients from particular regions or age groups). This could affect the generalizability of the findings to different populations. Therefore, future studies should include larger and more diverse samples to enhance the universality and accuracy of the model. Interpretability and Operability of the Model: Although the machine learning models constructed in this study demonstrate high accuracy and stability in predicting cognitive dysfunction, their “black-box” nature makes them less interpretable. Clinicians may find it difficult to fully understand and operate these models, especially when dealing with complex biomarkers and clinical indicators. Future research should focus on improving the interpretability of the models, providing more actionable guidance that can be integrated with clinical practices.

## Conclusion

In summary, a horizontal comparison of the above predictive models provides a deeper understanding of the application of machine learning in predicting cognitive dysfunction in LA patients. There is potential for further exploration of the clinical applications of these models and a comprehensive analysis of the underlying mechanisms of cognitive dysfunction diagnosis in LA patients. This study, through exploring the risk factors of cognitive dysfunction in LA patients and establishing a predictive model, aims to early identify high-risk groups for cognitive dysfunction in LA patients, guiding clinical interventions for early risk factors and reducing the disability rate caused by cognitive dysfunction.

## Data Availability

The raw data supporting the conclusions of this article will be made available by the authors, without undue reservation.
